# Efficacy of Plant Growth-Promoting Bacteria *Bacillus cereus* YN917 for Biocontrol of Rice Blast

**DOI:** 10.3389/fmicb.2021.684888

**Published:** 2021-07-19

**Authors:** Hu Zhou, Zuo-hua Ren, Xue Zu, Xi-yue Yu, Hua-jun Zhu, Xiao-juan Li, Jie Zhong, Er-ming Liu

**Affiliations:** ^1^College of Plant Protection, Hunan Agricultural University, Changsha, China; ^2^Hunan Provincial Key Laboratory for Biology and Control of Plant Diseases and Insect Pests, Changsha, China; ^3^Southern Regional Collaborative Innovation Center for Grain and Oil Crops in China, Changsha, China; ^4^Hunan Academy of Agricultural Sciences, Institute of Plant Protection, Changsha, China

**Keywords:** *Magnaporthe oryzae*, *Bacillus cereus*, biological control, plant growth promoting, antagonistic activities

## Abstract

*Bacillus cereus* YN917, obtained from a rice leaf with remarkable antifungal activity against *Magnaporthe oryzae*, was reported in our previous study. The present study deciphered the possible biocontrol properties. YN917 strain exhibits multiple plant growth-promoting and disease prevention traits, including production of indole-3-acetic acid (IAA), ACC deaminase, siderophores, protease, amylase, cellulase, and β-1,3-glucanase, and harboring mineral phosphate decomposition activity. The effects of the strain YN917 on growth promotion and disease prevention were further evaluated under detached leaf and greenhouse conditions. The results revealed that *B. cereus* YN917 can promote seed germination and seedling plant growth. The growth status of rice plants was measured from the aspects of rice plumule, radicle lengths, plant height, stem width, root lengths, fresh weights, dry weights, and root activity when YN917 was used as inoculants. YN917 significantly reduced rice blast severity under detached leaf and greenhouse conditions. Genome analysis revealed the presence of gene clusters for biosynthesis of plant promotion and antifungal compounds, such as IAA, tryptophan, siderophores, and phenazine. In summary, YN917 can not only be used as biocontrol agents to minimize the use of chemical substances in rice blast control, but also can be developed as bio-fertilizers to promote the rice plant growth.

## Introduction

Rice blast is considered as a devastating disease of rice plants caused by *Magnaporthe oryzae* (Miah et al., 2017). The disease has been reported throughout the world wherever rice is intensively cultivated including Europe (Isaac et al., 2010), Africa (Mgonja et al., 2016), Central America (Farman et al., 2017), Oceania, India (Jagadeesh et al., 2018), and China (Xu et al., 2019).

At present, the main measures to prevent and cure rice blast are breeding blast-resistant varieties, changing cultivation patterns (Mottaleb et al., 2019), and applying chemical fungicides. Breeding blast-resistant cultivars has proven to be the most effective, economical, and environment-friendly strategy for disease control, but there are restrictions such as long breeding cycles and variation of physiological races that might result in loss of blast resistance. The breeding of resistant varieties has been proven to be the most effective and economical strategy to control the rice blast, but the long breeding cycle and the loss of resistance due to the variation of physiological races are limiting factors. Up to now, the control of rice blast primarily relies on chemical fungicide, such as tricyclazole, isoprothiolane, tetrachlorobenzene, ediphenphos, blastin, carbendazim, and strobilurin (Wu et al., 2014). Although chemicals are widely available, the excessive dependence on chemical fungicides will cause high fungicide residues and environmental pollution, which is not conducive to the sustainable development of rice agriculture. Microbial fungicides can not only reduce the occurrence of diseases, but also maintain the ecological equilibrium (Zhou et al., 2019). Previous studies have reported that *Bacillus cereus* strains exhibited antifungal activity against *M. oryzae in vitro* or *in vivo*, which is expected to be developed as fungicides to control rice blast, such as the *B. cereus* HS24 (Huang et al., 2020), *B. cereus* AR156 (Jiang et al., 2020), and *B. cereus* KF822666 (Hassan et al., 2019). These studies provided a background for the development of biocides.

In our previous study, a bio-control strain of *B. cereus* YN917 (16S rRNA GenBank: MT990515.1) was isolated from a healthy rice leaf sample of the susceptible rice cultivar Xiangzaoxian No. 24 and stored in China Center for Type Culture Collection, Wuhan, China (CCTCC No. M2020655) (Zhou et al., 2021). To explore the biocontrol potential of strain YN917, this strain was investigated for plant growth-promoting and disease prevention traits, such as the secretion of IAA, ACC deaminase, siderophores, and enzymes, as well as decomposition of mineral phosphate and potassium. The biocontrol effects of strain YN917 in controlling rice blast and growth-promoting properties on rice under detached leaf and greenhouse conditions were evaluated. Moreover, its genomic properties such as the genes associated with production of various antifungal and plant growth-promoting compounds are described.

## Results

### Plant Growth-Promoting Activities

**FIGURE 1:**
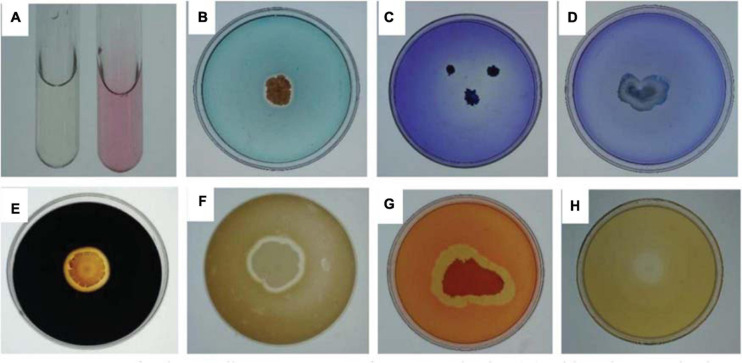
Assays for the *Bacillus cereus* YN917 for IAA production **(A)**. Siderophore production **(B)**. Phosphate solubilization activity **(C)**. Potassium solubilization activity of YN917 **(D)**. Amylase production **(E)**. Protease production **(F)**. Cellulase production **(G)**. β-1,3-glucanase production **(H)**.

**FIGURE 2:**
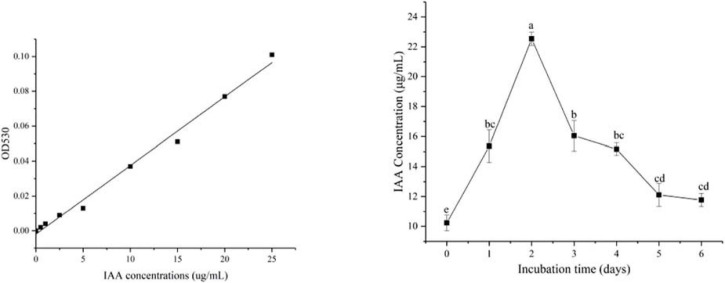
Levels of IAA produced by *B. cereus* YN917 under shaking conditions.

Indole acetic acid (IAA) production in the *B. cereus* YN917 was determined qualitatively and quantitatively. YN917 was confirmed to produce IAA through the pink chromogenic reaction (Figure 1A), and the IAA production seemed to be high according to the pink color (Table 1). The IAA production of YN917 was further quantified by the calculated standard curve equation y = 0.0039x-0.0019 (*R*^2^ = 0.9913, y is the absorbance and x is IAA concentration). YN917 showed the maximum 22.54 μg/ml IAA content at the second day of incubation (Figure 2). These results suggest that *B. cereus* YN917 could produce IAA even in the absence of exogenous tryptophan.

**TABLE 1:** Rice seed growth-promoting effects of *Bacillus cereus* YN917.

**Treatments**	**Germination (%)**	**Plumule length (cm)**	**Radicle length (cm)**
Control	91.33 ± 0.99 a	0.65 ± 0.14 b	0.97 ± 0.15 b
LB	92.00 ± 0.73 a	0.72 ± 0.03 b	1.08 ± 0.10 b
YN917	91.67 ± 1.21 a	1.31 ± 0.16 a	1.72 ± 0.21 a

*Control, LB, and YN917 are the treatments where the rice seedlings were treated with water, LB, and YN917, respectively. The germination, plumule length, and radicle length are represented as the means ± SE of at least three independent experiments. Different letters in each column indicate that the differences are significant (p < 0.05) using Duncan’s multiple range test.*

Spectrophotometric analysis revealed that strain YN917 grows better on ADF medium when compared with the control DF medium. These results suggested that YN917 has the ability to produce ACC deaminase.

Several traits of YN917 involved in plant growth promotion and disease prevention were tested *in vitro* (Figure 1). After 7 days of incubation at appropriate temperature, clear and distinct hydrolytic halos were individually formed around the YN917 colonies that were grown on solid medium containing CAS (Figure 1B), Ca_3_(PO_4_)_2_ (Figure 1C), soluble starch (Figure 1E), skimmed milk (Figure 1F), CMC (Figure 1G), and glucanase (Figure 1H). Therefore, it was suggested that *B. cereus* YN917 could secrete siderophore, amylase, protease, cellulase, and β-1, 3-glucanase, as well as have the potential to decompose mineral phosphate. However, there are obvious hydrolytic halos formed around YN917 colonies, which indicated that YN917 has no potential to solubilize potassium (Figure 1D).

### Growth-Promoting Efficacy of *B. cereus* YN917

The growth-promoting efficacy of YN917 on rice seed were examined, and the results are shown in Table 1. It revealed that endophytic bacterial YN917 displayed a growth-promoting effect on seed growth, significantly increasing plumule and radicle lengths (*p* > 0.05), but had no significant effect on germination rate. The average plumule length was 1.31 ± 0.16 cm, which was equivalent to an increase by 101.54% relative to the control when YN917 was inoculated. Meanwhile, it gave rise to the average radicle length of 1.72 ± 0.21 cm, which was equivalent to a 77.32% increase compared with the control. After inoculation with YN917 for 30 days, the growth parameters of rice seedlings (plant height, root length, stem circumference, root length, fresh weight, dry weight, and root activity) were significantly improved relative to control seedlings (Table 2). The quantitative results showed that the plant height, stem circumference, root length, fresh weight, dry weight, and root activity of rice seedlings treated with YN917 increased by 49.67, 17.39, 159.46, 30.29, 35.71, and 56.75%, respectively, when compared with those treated only by water. LB medium also improved rice plant growth, but the efficiency was minor and not significant. Overall, these results revealed that *B. cereus* YN917 enabled the inoculated rice seedlings to grow better than the non-inoculated control rice seedlings.

**TABLE 2:** Growth parameters of rice plants noted 30 days after their inoculation with *Bacillus cereus* YN917 compared with control.

**Treatments**	**Plant height (cm)**	**Steam width (mm)**	**Root length (cm)**	**Fresh weight (g)**	**Dry weight (g)**	**Root activity**
Control	22.65 ± 1.95 b	4.83 ± 0.02 c	2.98 ± 0.04 b	1.75 ± 0.05 c	0.70 ± 0.02 a	59.40 ± 1.14 b
LB	25.86 ± 0.98 b	5.04 ± 0.01 b	3.40 ± 0.13 b	1.83 ± 0.08 b	0.78 ± 0.02 a	63.13 ± 1.40 b
YN917	33.69 ± 1.27 a	5.67 ± 0.02 a	7.68 ± 0.35 a	2.28 ± 0.10 a	0.95 ± 0.03 a	93.11 ± 1.57 a

*Control, LB, and YN917 are the treatments where the rice seedlings were treated with water, LB, and YN917, respectively. Data represent the means ± SD from 3 independent replicates. Different letters in each column indicate that the differences are significant (p < 0.05) using Duncan’s multiple range test.*

### Biocontrol Efficacy of *B. cereus* YN917

**FIGURE 3:**
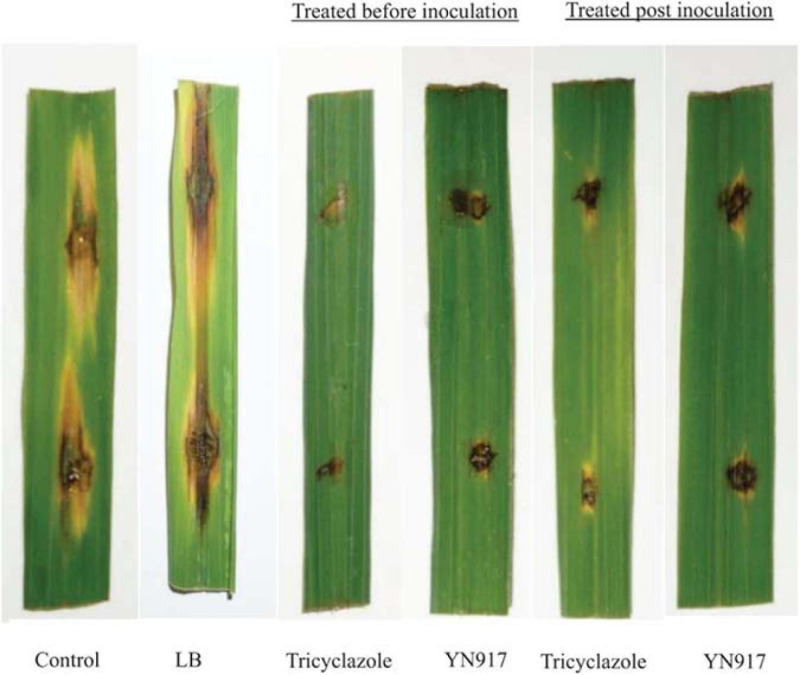
Lesion development 5 days after inoculation with strain *Bacillus cereus* YN917 following protective and therapeutic droplet treatments.

The biocontrol efficacy of *B. cereus* YN917 against *M. oryzae* was assayed on detached leaves and in pot experiments. The biocontrol efficacy determined in the detached leaf assays is shown in Table 3. Both for the prevention and treatment of disease, strain YN917 showed good anti-blast activity. In the prevention experiments, leaves treated with YN917 and 20% tricyclazole WP developed significant smaller lesions than leaves treated with water or LB (Figure 3). In the curative experiments, the leaf lesions were reduced when treated with YN917. Similar results were obtained in the disease treatment experiments. Furthermore, leaves treated with LB showed the longest rice blast lesions.

**TABLE 3:** Incidence rate of leaf blast development 5 days after inoculation with strain *Bacillus cereus* YN917 following protective and therapeutic droplet treatments.

**Treatments**	**Treated before inoculation**	**Treated after inoculation**
Control	98.00 ± 1.15 a	95.67 ± 2.33 a
LB	97.50 ± 2.50 a	96.00 ± 2.31 a
Tricyclazole	9.50 ± 0.76 b	15.33 ± 1.76 b
YN917	13.83 ± 1.30 b	20.67 ± 2.60 b

*Control, LB, tricyclazole, and YN917 are the treatments where the rice seedlings were treated with water, LB, 20% tricyclazole, and YN917, respectively. Data represent the means ± SD from 3 independent replicates. Different letters in each column indicate that the differences are significant (p < 0.05) using Duncan’s multiple range test.*

The results of the biocontrol efficacy in pot experiments are shown in Table 4. Compared with the treatments of 40% isoprothiolane WP or YN917 fermentation broth, the untreated rice seedlings showed the most serious rice blast, with a disease index of 60.04%. In the prevention experiments, rice seedlings treated with YN917 had a disease index of 18.68%, which was similar to that treated with tricyclazole and significantly lower than that treated with isoprothiolane (33.67%). Disease treatment experiments showed that the disease index of YN917 treatments was 20.65%, which was significantly lower than that of the isoprothiolane treatment (34.88%).

**TABLE 4:** Disease index development 7 days after inoculation with strain *Bacillus cereus* YN917 following protective and therapeutic spray treatments.

**Treatments**	**Treated before inoculation**			**Treated after inoculation**		
	**Disease incidence (%)**	**Disease index**	**Efficacy (%)**	**Disease incidence (%)**	**Disease index**	**Efficacy (%)**
Control	89.49 ± 3.38 a	58.65 ± 4.81 a	–	96.73 ± 0.58 a	60.04 ± 2.88 a	–
LB	93.41 ± 3.33 a	52.27 ± 4.06 a	–	91.81 ± 2.70 a	56.16 ± 3.74 a	–
Tricyclazole	58.65 ± 0.68 b	13.79 ± 1.00 c	76.49	60.60 ± 9.32 b	14.12 ± 3.81 c	76.48
YN917	61.52 ± 5.00 b	18.68 ± 3.21 c	68.15	61.17 ± 3.67 b	20.65 ± 3.18 c	65.61

*Control, LB, tricyclazole, and YN917 are the treatments where the rice seedlings were treated with water, LB, 20% tricyclazole, and YN917, respectively. Data represent the means ± SD from 3 independent replicates. Different letters in each column indicate that the differences are significant (p < 0.05) using Duncan’s multiple range test.*

### Analysis of the Genome Sequence

Because *B. cereus* YN917 was able to produce IAA, ACC-deaminase, and siderophores and solubilize inorganic phosphate and phosphate, to obtain insights into the mechanisms of antifungal and growth-promoting properties of *B. cereus* YN917, the genomic information of *B. cereus* YN917 was analyzed.

The complete genome sequence of strain YN917 (the GenBank accession number is IPRJNA687285 for the BioProject) includes multiple genes taking part in the synthesis of secondary metabolites and organic compounds that contribute to the antifungal activities against pathogens and plant growth promotion. According to the genomic annotation, the *phz*, *pcn*, and *hcn* gene clusters, which are associated with production of antifungal compounds, such as phenazine, were predicted in the YN917 genome. The 2-oxoglutarate dehydrogenase *sucA*, tryptophan 2,3-dioxygenase *TDO2*, arylformamidase *kynB*, kynureninase *kynU*, acyltransferase *atoB*, and amidase amiE, which are associated with hydrolyzing indole acetamide to indoleacetic acid, were predicted in the KEGG pathway of tryptophan metabolism of YN917 genome. The indole-3-glycerol phosphate synthase, *trpC*, tryptophan synthase beta chain, *trpB*, and tryptophan synthase alpha chain, *trpA*, which are associated with tryptophan, synthesize by themselves and several enzymes related to tryptophan synthesis were predicted in the YN917 genome. The genome also contains *pstSCAB-phoU* and beta-glucosidase genes that are involved in plant growth.

Six genes related to siderophore were found in ABC transporter pathway of *B. cereus* YN917, and five genes were found in porphyrin and chlorophyll metabolism, which are associated with the synthesis of these molecules as well as with the interaction and transport of iron, including iron complex transport system ATP-binding protein, iron complex transport system permease protein, iron complex transport system substrate-binding protein, iron (III) transport system ATP-binding protein, iron (III) transport system permease protein, iron (III) transport system substrate-binding protein, protoporphyrinogen coproporphyrinogen III oxidase, and Fe-coproporphyrin III decarboxylase. Genes related to siderophore have been identified, including *entA*, *entB*, *entC*, and *entE*, and genes related to iron metabolism regulated by IdeR were also identified, including *nuoA*, *nuoB*, *nuoC*, *nuoD*, *nuoH*, *nuoI*, *nuoJ*, *nuoK*, *nuoL*, *nuoM*, and *nuoN*. Overall, strain YN917 has a great potential as a biocontrol agent in rice cultivation.

## Discussion

The biocontrol activity of *Bacillus* spp. against rice blast has been confirmed in previous studies. Compared with the application of fungicides, the biological control of rice blast has the advantages of a low cost and environmental friendliness. In this study, the anti-blast activity of *B. cereus* YN917 was evaluated under detached and greenhouse conditions. The results indicated that strain YN917 was a potential biocontrol agent against rice blast. Notably, in a pot experiment, prevention treatment had a lower disease index than curative treatment; therefore, spraying YN917 in advance is the most effective measure to prevent rice blast. In general, biocontrol strain can induce plant systemic resistance by themselves (Tahir et al., 2017). On the other hand, it was evident that *B. cereus* YN917 efficiently promoted rice growth.

The excellent bio-control effects of YN917 could result from the production of bioactive secondary metabolites or compounds with antimicrobial properties. YN917 produced IAA, ACC deaminase, siderophores, and phosphate-solubilizing and major enzymes such as protease, amylase, cellulase, and β-1,3-glucanase. YN917 showed the production of IAA and ACC deaminase, an important trait of plant growth-promoting microorganisms (Velmurugan et al., 2015). IAA is a phytohormone, which regulates the growth of plant roots by stimulating the proliferation and elongation of root cells, but has no obvious role in bacteria itself (Lwin et al., 2012). ACC is a precursor for ethylene, one of the most important regulatory hormones of plant growth (Bernard, 2005; Glick, 2014; Torbaghan et al., 2016). ACC deaminases can synergistically interact with IAA and enhance plant growth.

In addition, strain YN917 showed the ability to produce siderophores. The production of siderophore is one of the most vital mechanisms to prevent the plant pathogens (Etesami and Maheshwari, 2018). In this study, the ability of YN917 to inhibit mycelium growth of *M. oryzae* was probably associated with the production of siderophore, and further analysis by antiSMASH revealed that a cluster involved in siderophore could be found. Iron is indispensable for DNA synthesis and respiration in organisms. However, the bioavailability of iron in the natural environment is limited by its low solubility of Fe^3+^ in soil. Siderophore is strong enough to remove iron from the environment and convert it into available substances for microbial cell growth (Sasirekha and Shivakumar, 2016). Antagonistic microorganisms could promote their colonization in plants by producing siderophores, as well as assist in obtaining iron nutrition, and limit and inhibit the growth of plant pathogens through iron competition (Eljounaidi et al., 2016).

Generally, bio-control microorganisms can interact with phytopathogens through a series of mechanisms, including the production of antibiotics and lytic enzymes, competition for nutrients, and ecological niches (Berg and Hallmann, 2006). In a previous study, Li et al. (2018) has reported that producing cellulase ability in antagonistic bacteria is essential for entrancing into the plant tissues. YN917 could be developed as a fungicide due to its ability to produce enzymes, which can provide an environment-friendly alternative method to safeguard from phytopathogens.

Genome analysis revealed that the genome of strain YN917 includes the genes associated with antifungal activities and plant growth promotion, such as phenazine, IAA, tryptophan, and siderophore. The genome sequence of *B. cereus* YN917 will contribute to elucidation of genetic mechanisms of the disease prevention and plant growth promotion.

## Conclusion

In the current study, we investigated a biocontrol strain that could be used as an alternative agent for controlling rice blast, and the experiment results also enhanced our comprehensive understanding toward the possible antifungal mechanisms of *B. cereus* YN917. It has potential to be developed as bio-agents and as bio-fertilizers due to their capabilities in the production of IAA, ACC deaminase, siderophore, and enzymes. The complete genome sequence and annotation of YN917 are summarized, and its genomic properties like the genes associated with production of antifungal activities and plant growth-promoting compounds are described.

## Materials and Methods

### Materials

*B. cereus* strain YN917 was maintained in 50% glycerol and stored at –80°C. Unless otherwise specified, the strain was grown on Luria–Bertani medium at 28°C (Lagier et al., 2015). *B. cereus* YN917 was grown on LB medium at 28°C, 180 rpm for 12 h, and the culture was diluted by sterile distilled water and adjusted to an optical density OD 600 = 1 before inoculation.

*Magnaporthe oryzae* was previously isolated and preserved by our laboratory, and maintained in potato dextrose agar (PDA) plates. Suspension was collected from 7-day-old cultures grown on PDA by washing with water, and then these were transferred to new tomato oatmeal agar plates cultured for 7 day to produce spores. The spores were washed off the plates with 0.1% (v/v) Tween 20 in sterile distilled water and adjusted to 10^5^ conidia/ml using a hemocytometer.

The seeds used in this study were Lijiangheituanxingu (Japonica cultivar) and indica cultivar Xiangwanxian No. 12 (Indica rice cultivar; Gold Non-gfeng Seed Industry Technology Co., Ltd., China), which were susceptible to rice blast and were used as the test rice varieties. The seeds were provided by Hunan Academy of Agricultural Sciences, Changsha, Hunan, China.

The commercial fungicide used in this study was 20% tricyclazole WP (750 times dilution; Jiangsu Changqing Agrochemical Co., Ltd., China).

### Plant Growth Promotion Assays

#### Indole-3-Acetic Acid (IAA) Production

Qualitative assay of IAA production was determined by the method described by Bano and Musarrat (2003). *B. cereus* YN917 was inoculated in LB medium and incubated in a shaker (180 rpm, 28°C) for 6 days. One milliliter of the culture was mixed with 2 ml of Salkowski reagent. Appearance of pink color indicates IAA production. The non-inoculated broth medium was used as a control. All treatments consisted of three replicates and were repeated twice.

For quantitative assay of IAA production, a 5-ml aliquot was withdrawn periodically from each culture flask at 0, 1, 2, 3, 4, 5, and 6 days, respectively, and centrifugated (10,000 rpm for 5 min). After that, 2 ml of YN917 supernatant was mixed with 2 drops of phosphoric acid and 4 ml Salkowski’s reagent. The optical density of per sample was measured at 530 nm and the amount of YN917 IAA produced was calculated by comparing with the standard IAA curve.

#### 1-Aminocyclopropane-1-Carboxylate (ACC) Deaminase Production

The 1-aminocyclopropane-1-carboxylic acid deaminase activity of *B. cereus* YN917 was detected by using DF medium and ADF medium (Beijing Baiaolaibo Technology Co., Ltd., China) (Dworkin and Foster, 1958; Elżbieta and Stanisław, 2015; Chandra and Sharma, 2016). These mediums were inoculated with 1% (v/v) fresh YN917 cultures and incubated in a shaker (180 rpm, 28°C) for 5 days. The optical density was measured at 600 nm by a spectrophotometer from three replicates; it was considered positive for ACC deaminase production for YN917 that grew in ADF medium and showed no growth on DF medium.

#### Siderophore Production

Chrome Azurol-S (CAS) detection medium (Qingdao Hope Bio-Technology Co., Ltd., China) was used to qualitatively analyze the YN917 siderophore production (You et al., 2005). These plates were spot inoculated with YN917 and kept at 28°C for 7 days. A yellow-orange halo around YN917 colony was considered as an indicator of siderophore production. The non-inoculated plates were used as control.

#### Phosphate Solubilization

Pikovskaya (PVK) agar plates were used to qualitatively determine the YN917 insoluble organic phosphate solubilization (Sunita, 2016). The medium consists of 10 g glucose, 0.5 g yeast extract, 0.5 g ammonium sulfate (NH_4_)_2_SO_4_, 0.2 g NaCl, 0.2 g KCl, 0.1 g MgSO_4_⋅7H_2_O, 2 mg MnSO_4_, 2 mg FeSO_4_⋅7H_2_O, 25 mg bromophenol blue, 20 g agar, and 0.5% tricalcium phosphate Ca_3_(PO_4_)_2_ in 1 L of distilled water. These plates were spot inoculated with YN917 and kept at 28°C for 7 days. The clear halo around YN917 colony indicated positive phosphate solubilization. The non-inoculated plates were used as control.

#### Potassium Solubilization

Potassium feldspar agar plates were used to qualitatively determine the YN917 potassium-solubilizing capability. The medium consists of 5 g sucrose, 5 g potassium feldspar powder, 2 g Na_2_HPO_4_, 0.5g MgSO_4_⋅7H_2_O, 0.1 g CaCO_3_, 5 mg FeCl_3_, 25 mg bromophenol blue, and 20 g agar in 1 L of distilled water, pH 7.2, inoculated with YN917, and incubated at 28°C for 7 days. The clear halo around YN917 colony on potassium feldspar agar plates was considered indicative of potassium solubilization. The non-inoculated plates were used as control.

#### Assay for Enzyme Production

Protease production was determined on skimmed milk LB agar medium (10 g tryptone, 10 g NaCl, 5 g yeast extract, 20 g agar, and 10% sterile skimmed milk in 1 L distilled water, pH 7.4). A positive reaction was read after 2–7 days of incubation at 28°C, with the formation of halo zone surrounding YN917 colonies indicating positive result for protease production. The non-inoculated plates were used as control.

Amylase production was determined by soluble starch agar medium (10 g peptone, 5 g yeast extract, 2 g soluble starch, and 20 g agar in 1 L distilled water, pH 7.0). After 2 days of incubation at 28°C, the ability of YN917 to hydrolyze amylase was determined by the appearance of a halo zone around the colonies and confirmed by Lugol’s iodine solution for 15 min and 70% ethanol. The non-inoculated plates were used as control.

Cellulase production was determined by the carboxymethyl cellulose (CMC) agar (10 g peptone, 10 g yeast extract, 10 g CMC, 5 g NaCl, 1 g KH_2_PO_4_, and 20 g agar in 1 L distilled water, pH 7.0). After 5 days of incubation at 28°C, the ability of isolates to hydrolyze cellulose was determined by the appearance of a clear zone around the colonies and confirmed after pouring 0.1% Congo Red solution into 1 M NaCl (Thomas et al., 2018). The non-inoculated plates were used as control.

β-1,3-Glucanase production was determined by medium (peptone 10 g, yeast extract 5 g, NaCl 10 g, Congo Red 0.04 g, and agar 20 g in 1 L distilled water, pH 7.0). A positive reaction was read after 7 days of incubation at 28°C, with the formation of halo zone surrounding YN917 colonies indicating a positive result for β-1,3-glucanase production (Srividya et al., 2012). The non-inoculated plates were used as control.

### Evaluation of Plant Growth-Promoting Ability of *B. cereus* YN917

#### Rice Seed Sterilization

Healthy seeds of rice variety Xiangzaoxian No. 24 were selected and disinfected by a 2-min wash in 75% alcohol, and rinsed three times in sterile distilled water (Oyebanji et al., 2009).

#### Seed Germination Assay

The sterilized seeds were completely soaked in 20 ml YN917 suspension for 12 h to make YN917 colonize on the rice seeds, followed by three rinses of sterile distilled water, and then transferred to Petri plates containing two water-soaked filter papers (50 seeds per replicate) at 28°C for germination. Seeds soaked in LB medium or distilled water served as un-inoculated controls. Germination percentage was recorded at the second day. Rice plumule and radicle lengths were measured at the third day.

### Pot Experiment

The plant growth-promoting effects of *B. cereus* YN917 were investigated on Xiangzaoxian No. 24 in the greenhouse. For the pot experiment, the surface-sterilized rice seeds with normal and healthy appearance were selected and planted to every pot (20 cm × 15 cm × 10 cm) filled with field soil and kept in the cultivation greenhouse. The planting soil was taken from Liuyang River in Hunan Agricultural University, Hunan Province, China, and used after disinfection under 121°C for 15 min. Seedlings were grown in natural conditions and irrigated daily. Meanwhile, each pot was irrigated with 50 ml YN917 suspension or water or LB medium once every 10 days. The experiment was conducted three times. Planting for 30 days, the seedlings of each treatment were collected for detection of plant height, root length, stem circumference, fresh weight, and dry weight at the same time.

### Biocontrol Assays

#### Antifungal Activity on Detached Rice Leaves

The biocontrol effect of *B. cereus* YN917 was tested by using punch inoculation on rice leaves of Lijiangheituanxingu which is a rice blast–susceptible variety. Fresh leaves (5-leaf stage) about 7 cm were cut from rice plants. To reinforce the inoculation, a sterilized needle was used to slightly scratch but not puncture the leaf surface. Then 5 μl conidial suspension was added to the wounds, and 5 μl of YN917, sterile water (negative control), LB, or 750 times dilution of 20% tricyclazole WP was added to inoculation points at 24 h before inoculation, as protective test (or 24 h post-inoculation, therapeutic test). The treated materials were placed to 85–100% relative humidity and temperature is 28°C with a 12-h photoperiod. Lesion length was measured at the seventh day after inoculation. All treatments were repeated three times independently (Kunova et al., 2013).

#### Antifungal Activity in Greenhouse

The biocontrol effect of *B. cereus* YN917 on rice blast was studied by leaf-spraying inoculation with Xiangzaoxian No. 24. When the rice seedlings at the 3-leaf stage were challenge inoculated with conidia suspension (5 ml/pot), 5 ml of YN917 fermentation broth, 20% tricyclazole WP LB, or sterile water was sprayed at 24 h before inoculation, as protective test (or 24 h post-inoculation, therapeutic test). Completely randomized design (CRD) with three replications was used to conduct the experiment. These experiments were carried out in two independent batches. Rice seedlings were incubated in a moist chamber with a 12-h photoperiod, with temperatures ranging 25–32°C. The disease index (DI) was calculated according to the Standard Evaluation System (SES) for International Rice Research Institute (IRRI) (Marta et al., 2011) as follows at 7 days post-inoculation by collecting 50 rice leaves randomly.

### Genome Sequencing and Analysis

*B. cereus* YN917 genomic DNA was extracted using CTAB method, and whole genome sequencing was performed using the PacBio platform and Illumina HiSeq platform at Majorbio Bio-Pharm Technology Co., Ltd. (Shanghai, China). Six databases were used for general function annotation: GO (Gene Ontology) (Moura et al., 2009), KEGG (Kyoto Encyclopedia of Genes and Genomes) (Kanehisa and Goto, 2000), COG (Clusters of Orthologous Groups) (Juhl et al., 2008), NR (Non-Redundant Protein database) (Hossain et al., 2018), Swiss-Prot (Bairoch and Apweiler, 2000), and Pfam (Mistry et al., 2021).

### Data Analysis

All statistical analysis was performed using IBM SPSS Statistics 20.0 software with one-way ANOVA. All values are expressed as the mean ± SD of at least three independent experiments.

## Data Availability Statement

The datasets presented in this study can be found in online repositories. The names of the repository/repositories and accession number(s) can be found below: NCBI BioProject, accession no: PRJNA687285.

## Author Contributions

HZ and Z-HR designed the experiment. HZ, XZ, X-YY, and H-JZ performed the experiment. X-JL, Z-HR, and E-ML contributed reagents and materials. HZ, Z-HR, JZ, and E-ML wrote the manuscript. All authors contributed to the article and approved the submitted version.

## Conflict of Interest

The authors declare that the research was conducted in the absence of any commercial or financial relationships that could be construed as a potential conflict of interest.

## References

[B1] Bairoch A. Apweiler R. (2000). The SWISS-PROT protein sequence database and its supplement TrEMBL in 2000. *Nucleic Acids Res.* 28 45–48. 10.1093/nar/28.1.45 10592178 PMC102476

[B2] Bano N. Musarrat J. (2003). Characterization of a new *Pseudomonas aeruginosa* strain NJ-15 as a potential biocontrol agent. *Curr. Microbiol.* 46 324–328. 10.1007/s00284-002-3857-8 12732958

[B3] Berg G. Hallmann J. (2006). “Control of plant pathogenic fungi with bacterial endophyte,” in *Microbial Root Endophytes*, eds Schulz B. J. E. Boyle C. J. C. Sieber T. N. (Berlin: Springer) Vol. 9 53–70 10.1007/3-540-33526-9_4

[B4] Bernard R. G. (2005). Modulation of plant ethylene levels by the bacterial enzyme ACC deaminase. *FEMS Microbiol. Lett.* 251 1–7. 10.1016/j.femsle.2005.07.030 16099604

[B5] Chandra D. Sharma A. K. (2016). Isolation and characterization of plant growth promoting bacteria containing ACC deaminase from soil collected from central Himalayan region of Uttarakhand, India. *Int. J. Curr. Microbiol. Appl. Sci.* 5 436–445. 10.20546/ijcmas.2016.508.047

[B6] Dworkin M. Foster J. (1958). Experiments with some microorganisms which utilize ethane and hydrogen. *J. Bacteriol.* 75 592–603. 10.1128/jb.75.5.592-603.1958 13538930 PMC290115

[B7] Eljounaidi K. Lee S. K. Bae H. (2016). Bacterial endophytes as potential biocontrol agents of vascular wilt diseases-review and future prospects. *Biol. Control* 103 62–68. 10.1016/j.biocontrol.2016.07.013

[B8] Elżbieta G. M. Stanisław J. P. (2015). Various effects of fluorescent bacteria of the genus *Pseudomonas* containing ACC deaminase on wheat seedling growth. *Microbiol. Res.* 181 112–119. 10.1016/j.micres.2015.04.005 25983132

[B9] Etesami H. Maheshwari D. K. (2018). Use of plant growth promoting rhizobacteria (PGPRs) with multiple plant growth promoting traits in stress agriculture: action mechanisms and future prospects. *Ecotoxicol. Environ. Saf.* 156 225–246. 10.1016/j.ecoenv.2018.03.013 29554608

[B10] Farman M. Peterson G. Chen L. Starnes J. Valent B. Bachi P. (2017). The *Lolium* pathotype of *Magnaporthe* oryzae recovered from a single blasted wheat plant in the United States. *Plant Dis.* 101 684–692. 10.1094/pdis-05-16-0700-re 30678560

[B11] Glick B. R. (2014). Bacteria with ACC deaminase can promote plant growth and help to feed the world. *FEMS Microbiol. Rev.* 169 30–39. 10.1016/j.micres.2013.09.009 24095256

[B12] Hassan E. Hossein A. A. Hossein M. H. (2019). Root bacterial endophytes as potential biological control agents against fungal rice pathogens. *Arch. Phytopathol. Plant Prot.* 52 560–581. 10.1080/03235408.2018.1557884

[B13] Hossain M. U. Omar T. M. Alam I. Das K. C. Mohiuddin A. K. M. Keya C. A. (2018). Pathway based therapeutic targets identification and development of an interactive database CampyNIBase of *Campylobacter* jejuni RM1221 through non-redundant protein dataset. *PLoS One* 13:e0198170. 10.1371/journal.pone.0198170 29883471 PMC5993290

[B14] Huang W. Liu X. Zhou X. Wang X. Liu H. (2020). Calcium signaling is suppressed in *Magnaporthe* oryzae conidia by *Bacillus cereus* HS24. *Phytopathology* 110 309–316. 31556343 10.1094/PHYTO-08-18-0311-R

[B15] Isaac L. L. Didier T. Mercedes C. M. (2010). Evidence for rapid changes in the population genetic structure of *Magnaporthe* oryzae in Southern Spain. *J. Phytopathol.* 158 785–791. 10.1111/j.1439-0434.2010.01699.x

[B16] Jagadeesh D. Prasanna Kumar M. K. Devaki N. S. (2018). Population analysis of *Magnaporthe* oryzae by using endogenous repetitive DNA sequences and mating-type alleles in different districts of Karnataka, India. *J. Appl. Genet.* 59 365–375. 10.1007/s13353-018-0453-6 29971754

[B17] Jiang C. H. Fan Z. L. Li Z. J. Niu D. D. Li Y. Zheng M. Z. (2020). *Bacillus cereus* AR156 triggers induced systemic resistance against *Pseudomonas syringae* pv. *tomato* DC3000 by suppressing miR472 and activating CNLs-mediated basal immunity in *Arabidopsis.* *Mol. Plant Pathol.* 21 854–870. 10.1111/mpp.12935 32227587 PMC7214473

[B18] Juhl J. L. Philippe J. Michael K. Christian M. Jean M. Tobias D. (2008). eggNOG: automated construction and annotation of orthologous groups of genes. *Nucleic Acids Res.* 36(suppl. 1) 250. 10.1093/nar/gkm796 PMC2238944 17942413

[B19] Kanehisa M. Goto S. (2000). KEGG: kyoto encyclopedia of genes and genomes. *Nucleic Acids Res.* 28 27–30. 10592173 10.1093/nar/28.1.27 PMC102409

[B20] Kunova A. Pizzatti C. Cortesi P. (2013). Impact of tricyclazole and azoxystrobin on growth, sporulation and secondary infection of the rice blast fungus, *Magnaporthe* oryzae. *Pest Manage. Sci.* 69 278–284. 10.1002/ps.3386 22933369

[B21] Lagier J. C. Edouard S. Pagnier I. Mediannikov O. Drancourt M. Raoult D. (2015). Current and past strategies for bacterial culture in clinical microbiology. *Clin. Microbiol. Rev.* 28 208–236. 10.1128/cmr.00110-14 25567228 PMC4284306

[B22] Li D. X. Ni K. K. Zhang Y. C. Lin Y. L. Yang F. (2018). Influence of lactic acid bacteria, cellulase, cellulase-producing *Bacillus pumilus* and their combinations on alfalfa silage quality. *J. Integr. Agric.* 17 172–186.

[B23] Lwin K. M. Myint M. M. Tar T. Aung W. Z. M. (2012). Isolation of plant hormone (Indole-3-Acetic Acid-IAA) producing rhizobacteria and study on their effects on maize seedling. *Eng. J.* 16 137–144. 10.4186/ej.2012.16.5.137 12383539

[B24] Marta C. C. F. Gisele B. D. S. Valácia L. S. Márcio V. C. Alessandra J. G. M. Anne S. P. (2011). Leaf blast (*Magnaporthe oryzae*) suppression and growth promotion by rhizobacteria on aerobic rice in Brazil. *Biol. Control.* 58 160–166. 10.1016/j.biocontrol.2011.04.016

[B25] Mgonja E. M. Balimponya E. G. Kang H. X. Bellizzi M. Park C. H. Li Y. (2016). Genome-Wide association mapping of rice resistance genes against *Magnaporthe* oryzae Isolates from Four African Countries. *Phytopathology* 106 1359–1365. 10.1094/phyto-01-16-0028-r 27454702

[B26] Miah G. Rafifii M. Y. Ismail M. R. Sahebi M. Hashemi F. S. G. Yusuffff O. (2017). Blast disease intimidation towards rice cultivation: a review of pathogen and strategies to control. *J. Anim. Plant Sci.* 27 1058–1066.

[B27] Mistry J. Chuguransky S. Williams L. Qureshi M. Salazar G. A. Sonnhammer E. L. L. (2021). Pfam: the protein families database in 2021. *Nucleic Acids Res.* 49 412–419. 10.1093/nar/gkaa913 PMC7779014 33125078

[B28] Mottaleb K. A. Govindan V. Singh P. K. Sonder K. He X. Y. Singh R. P. (2019). Economic benefits of blast-resistant biofortified wheat in Bangladesh: the case of BARI Gom 33. *Crop Prot.* 123 45–58. 10.1016/j.cropro.2019.05.013 31481821 PMC6686726

[B29] Moura A. Soares M. Pereira C. Leitão N. Henriques I. Correia A. (2009). INTEGRALL: a database and search engine for integrons, integrases and gene cassettes. *Bioinformatics* 25 1096–1098. 10.1093/bioinformatics/btp105 19228805

[B30] Oyebanji O. B. Nweke O. Odebunmi O. Galadima N. B. Idris M. S. Nnodi U. N. (2009). Simple, effective and economical explant-surface sterilization protocol for cowpea, rice and sorghum seeds. *Afr. J. Biotechnol.* 8 5395–5399.

[B31] Sasirekha B. Shivakumar S. (2016). Siderophore production by *Pseudomonas aeruginosa* FP6, a biocontrol strain for *Rhizoctonia* solani and *Colletotrichum* gloeosporioides causing diseases in chilli. *Agric. Nat. Resour.* 50 250–256. 10.1016/j.anres.2016.02.003

[B32] Srividya S. Thapa A. Bhat D. V. Golmei K. Dey N. (2012). Streptomyces sp. 9p as effective biocontrol against chilli soil borne fungal pathogens. *Eur. J. Exp. Biol.* 2 163–173.

[B33] Sunita G. (2016). Phosphate dissolving fungi: mechanism and application in alleviation of salt stress in wheat. *Microbiol. Res.* 193 94–102. 10.1016/j.micres.2016.09.005 27825490

[B34] Tahir H. A. S. Gu Q. Wu H. Niu Y. Huo R. Gao X. (2017). Bacillus volatiles adversely affect the physiology and ultra-structure of *Ralstonia solanacearum* and induce systemic resistance in tobacco against bacterial wilt. *Sci. Rep.* 7:40481. 28091587 10.1038/srep40481 PMC5238454

[B35] Thomas L. Ram H. Singh V. P. (2018). Inducible cellulase production from an organic solvent tolerant *Bacillus* sp. SV1 and evolutionary divergence of endoglucanase in different species of the genus *Bacillus*. *Braz. J. Microbiol.* 49 429–442. 10.1016/j.bjm.2017.05.010 29157901 PMC5914138

[B36] Torbaghan M. E. Lakzian A. Astaraei A. R. Fotovat A. Besharati H. (2016). Quantitative comparison of ammonia and 3-indoleacetic acid production in halophilic, alkalophilic and haloalkalophilic bacterial isolates in soil. *J. Fundam. Appl. Sci.* 3 194–202.

[B37] Velmurugan A. Sakthivel K. Swarnam T. P. Rachael S. Roy D. S. (2015). Assessment of the plant growth promotion and phosphorus solubilization by rhizosphere bacteria isolated from Troporthents soils of Bay Island. *Trends Biosci.* 8 2888–2892.

[B38] Wu W. H. Wang L. Zhang S. Liang Y. Q. Zheng X. L. He C. P. (2014). Assessment of sensitivity and virulence fitness costs of the AvrPik alleles from *Magnaporthe* oryzae to isoprothiolane. *Genet. Mol. Res. GMR* 13 9701–9709. 10.4238/2014.november.24.1 25501181

[B39] Xu T. Cao L. D. Zeng J. R. Christopher M. M. F. Yang Y. Z. Hu X. C. (2019). Plant root exudates are involved in *Bacillus* cereus AR156 mediated biocontrol against *Ralstonia* solanacearum. *Front. Microbiol.* 10:98. 10.3389/fmicb.2019.00098 30766525 PMC6365458

[B40] You J. L. Cao L. X. Liu G. F. Zhou S. N. Tan H. M. Lin Y. C. (2005). Isolation and characterization of actinomycetes antagonistic to pathogenic *Vibrio* spp. From nearshore marine sediments. *World J. Microbiol. Biotechnol.* 21 679–682. 10.1007/s11274-004-3851-3

[B41] Zhou G. Y. Zhou Q. Zhu Y. H. (2019). The antifungal action mode of the rice endophyte *Streptomyces* hygroscopicus OsiSh-2 as a potential biocontrol agent against the rice blast pathogen. *Pestic. Biochem. Physiol.* 160 58–69. 31519258 10.1016/j.pestbp.2019.06.015

[B42] Zhou H. Zhu H. Hu L. Yu X. Ren Z. Liu E. (2021). Characterization and inhibitory effects of an antifungal protein from the *Bacillus* cereus strain YN917. *Int. J. Agric. Biol.* 25 1153–1160.

